# Therapeutic effect of induction therapy including nab-paclitaxel followed by surgical resection for the patients with locally advanced non-small-cell lung cancer

**DOI:** 10.1186/s13019-024-02955-w

**Published:** 2024-07-05

**Authors:** Hidetaka Uramoto, Nozomu Motono, Shun Iwai

**Affiliations:** Department of Thoracic Surgery, Kanazawa Medical University, 1-1 Daigaku, Uchinada-Machi, Kahoku-Gun, Ishikawa, 920-0293 Japan

**Keywords:** NSCLC, Induction therapy, Surgical resection, Therapeutic effect

## Abstract

**Background:**

Lung cancer is associated with a high mortality rate worldwide. Non-small-cell lung cancer (NSCLC) is a major subtype of lung cancer. Carboplatin (CBDCA) plus nab-paclitaxel (PTX) has become a standard treatment for advanced unresectable NSCLC. However, treatment with nab-PTX has not been established as a standard therapy for resectable locally advanced (LA)-NSCLC.

**Methods:**

We conducted a comprehensive study involving consecutive patients with locally advanced NSCLC who underwent induction therapy including nab-PTX followed by surgical resection. Fifteen patients with locally advanced NSCLC underwent induction therapy including nab-PTX followed by surgical resection. Concurrent chemoradiotherapy (CRT) consisted of weekly administration of nab-PTX (50 mg/m^2^) plus CBDCA (area under the plasma concentration time curve (AUC) 2) and thoracic radiotherapy (50 Gy/25 fractions).

**Results:**

The clinical stages were as follows: IIB (*n =*1), IIIA (*n =*12), and IIIC (*n =*2). Downstaging was observed in 73% (11/15) of patients on comparison with the clinical stage before concurrent CRT. Adverse drug reactions were observed in seven patients. Complete resection was performed in all patients. The re-evaluated pathological stage after pretreatment was diagnosed as stage 0 in three patients, stage IA1 in six, stage IA2 in one, and stage IIIA in five. The pathological effects of previous therapy were as follows: Ef3 (*n =*3), Ef2 (*n =*9), and Ef1a (*n =*3).

**Conclusion:**

The therapeutic effect of induction therapy including nab-PTX was promising. Induction CRT, including nab-PTX, followed by resection, may be a viable alternative treatment option for locally advanced NSCLC.

## Introduction

Lung cancer has a high mortality rate worldwide [1]. Approximately one-third of all LA-NSCLC patients have unresectable disease at the time of the diagnosis [2]. In this patient group, CRT, in addition to immune checkpoint inhibitors (ICIs), is the standard treatment for unresectable LA-NSCLC [3]. However, the median progression-free survival (PFS) has been reported to be 16.9 months [3]. Jang et al. examined the recurrence pattern of 171 NSCLC patients treated with concurrent CRT using real-world data. They reported 33 cases of locoregional failure among 89 patients treated with ICI consolidation following concurrent CRT [4]. These findings suggest the importance of local control in eradicating the disease.

The role of surgery in treating LA-NSCLC is controversial, and neoadjuvant therapy plays an important role in the treatment of this patient group [5–11]. Neoadjuvant therapy not only contributes to the regression of the primary tumor and metastatic lymph nodes, decreases the tumor stage, and ameliorates the surgical R0 resection rate but also removes micrometastases and reduces the risk of postoperative recurrence [12, 13]. Thus, induction CRT followed by surgery may be indicated in select patients with resectable LA-NSCLC [5–11]. However, there is no unified cytotoxic drug regimen, so paclitaxel (PTX) is commonly used.

The 130-nm albumin-bound formulation PTX (nab-PTX), which comprises nanoparticles of PTX bound to human serum albumin, is easier to use than traditional PTX [14]. Preclinical xenograft models have suggested that nab-PTX yields high maximal circulating and intratumoral concentrations of free PTX and increased antitumor activity [15]. There are additional advantages as well, in that it can be administered to patients who have hypersensitivity to alcohol or solvents while allowing for reduced injection times without requiring steroid prophylaxis [16]. In clinical trials, patients receiving CBDCA plus nab-PTX had a significantly higher objective response rate than patients receiving CBDCA plus PTX (33% vs. 25%; response rate ratio 1.313 [95% confidence interval 1.082–1.593]; p = 0·005), and the incidence of characteristic adverse events was significantly lower in the CBDCA plus nab-PTX group than in the CBDCA plus PTX group [14]. Based on these findings, CBDCA plus nab-PTX has become a standard treatment for advanced unresectable NSCLC [16, 17].

Given the above, we hypothesized that nab-PTX would be more effective than PTX as an advanced unresectable NSCLC drug for induction CRT followed by surgery for resectable LA-NSCLC. Currently, there are no reports on the use of nab-PTX for resectable LA-NSCLC. This study is thus the first to report on the effect of induction CRT including nab-PTX followed by surgical resection for resectable LA-NSCLC.

## Patients and methods

### Patients

All patients included in this study provided their written informed consent for treatment after receiving a comprehensive explanation of the observational research and privacy policy. This study was conducted in accordance with the principles of the Declaration of Helsinki and approved by the ethics committee of Kanazawa Medical University (approval no. C076).

Before initiating treatment, a pretreatment evaluation was conducted, which included a review of the patient’s medical history, a thorough physical examination, a complete blood cell count, and an analysis of serum chemistry data, including electrolyte levels, enzymes reflecting the liver function, bilirubin, creatinine, and coagulation values. Chest radiography, computed tomography (CT) of the chest and upper abdomen, and bronchoscopy were also performed. Preoperative cardiovascular risk assessment was conducted based on electrocardiography (ECG) and ultrasound cardiography (UCG). Clinical staging was performed using positron emission tomography (PET) and magnetic resonance imaging (MRI) of the brain [9]. Basically, lymph node positivity was judged only if EBUS-TBNA was pathologically positive for lymph node metastasis before treatment or if the short diameter of the lymph node was ≥ 1.0 cm and lymph node metastasis was suspected by PET-CT. The smoking history was assessed using the Brinkman Index (BI), which was calculated by multiplying the number of cigarettes smoked per day by the number of years the subject had been smoking.

### Treatment strategy

A comprehensive risk analysis was conducted preoperatively, and the decision to proceed with the operation was made collaboratively by a committee comprising an attending radiation oncologist, thoracic surgeon, medical oncologist, and pulmonologist. A post-induction re-evaluation was performed between six and eight weeks after induction therapy. Toxicity for all patients was assessed and graded using the National Cancer Institute Common Terminology Criteria for Adverse Events version 4.0. After a careful re-evaluation, surgery was attempted in cases in which R0 resection was considered feasible [18].

All surgical procedures were performed using either open thoracotomy or video-assisted thoracoscopic surgery (VATS). Systematic removal of mediastinal lymph nodes, including contiguous fatty tissue containing lymphatic vessels, was performed. We defined extended resection as a surgical operation that encompasses combined resection of adjacent structures along with complex lobectomy with subsequent bronchial reconstruction [18]. Complete resection was achieved based on a comprehensive review of both surgical and pathological findings, which indicated the absence of tumor cells in both the surgical margins and the highest mediastinal lymph node. In general, the bronchial stump is covered with pericardial fat tissue or intercostal muscle pedicle. Pericardial fat tissue or an intercostal muscle pedicle was thus used to cover the bronchial stump [19]. All surgical specimens were subjected to pathological analysis according to the 8th edition of the TNM classification, as outlined by the International Association for the Study of Lung Cancer (IASLC). The criteria specified in the eighth edition of the General Rules for Clinical and Pathological Records of Lung Cancer were used to assess the pathological effect of induction therapy: pathologically complete response (Ef3, indicating complete cancer cell death), major response (Ef2, signifying less than one-third of cancer cells remaining viable), minor response (Ef1, indicating that more than one-third of cancer cells remained viable), or no response (Ef0) [20]. To create a three-dimensional stacked area graph, we set stages 0, IA1, IA2, IA3, IB, IIA, IIB, IIIA, IIIB, IIIC, IVA, and IVB at 0, 1, 1.25, 1.5, 1.75, 2.25, 2.75, 3.15, 3.5, 3.75, 4.25, and 4.75, respectively. The vertical axis of the stacked area graph shows the total score when quantifying the stage.

### Postoperative management and follow-up

Postoperative management and follow-up procedures involved retrospective collection of data from all patients. These data encompassed a comprehensive patient history, age, sex, clinical and pathological staging, histological findings, treatment methods, and surgical details. After the surgery, the patient was discharged from the hospital. Follow-up data were collected from outpatient departments. In principle, the patients underwent a physical examination, chest roentgenography, analysis of blood chemistry data, and measurements of tumor marker levels every three months. Chest and abdominal CT, brain magnetic resonance imaging, and bone scintigraphy or positron PET were generally performed every six months until two years after surgery and then annually for five years or more [9]. Other assessments were conducted in response to the emergence of signs or symptoms indicating disease progression. The overall survival (OS) was defined as the time from the date of registration for initial treatment to the patient’s death or the date of the most recent follow-up examination. The recurrence-free survival (RFS) was defined as the time from registration until objective tumor recurrence or death.

## Results

### Patient characteristics

**Fig. 1:**
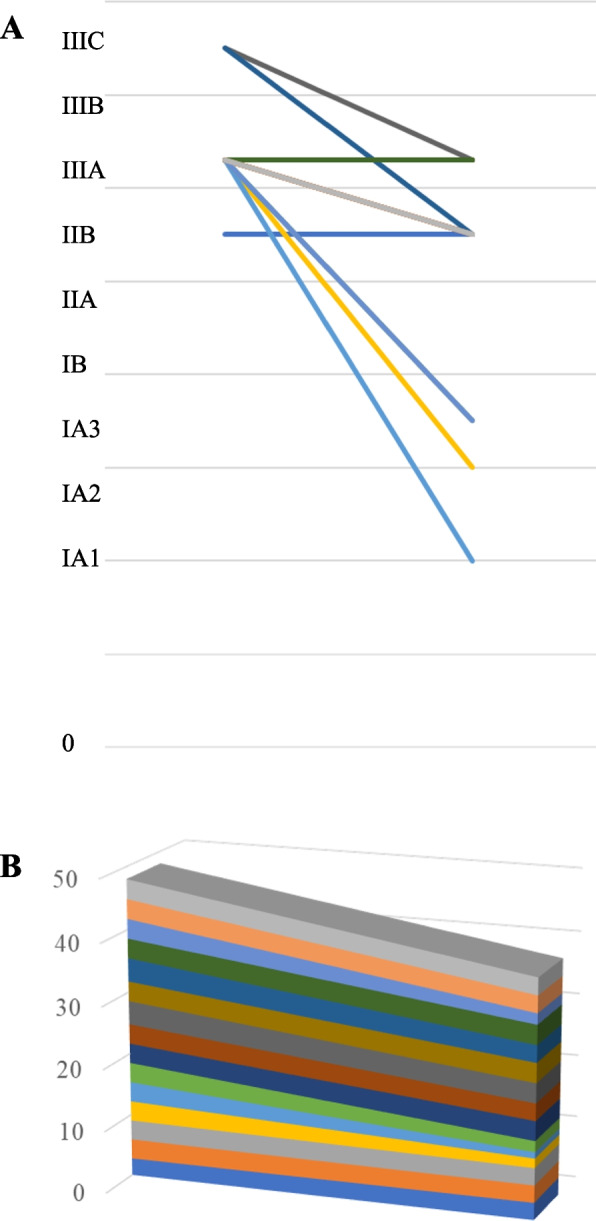
**A** The line chart of changes in the clinical stage and re-evaluated clinical stage after pretreatment (before CRT) in each patient. **B** A three-dimensional stacked area graph of changes in the clinical stage

**Fig. 2:**
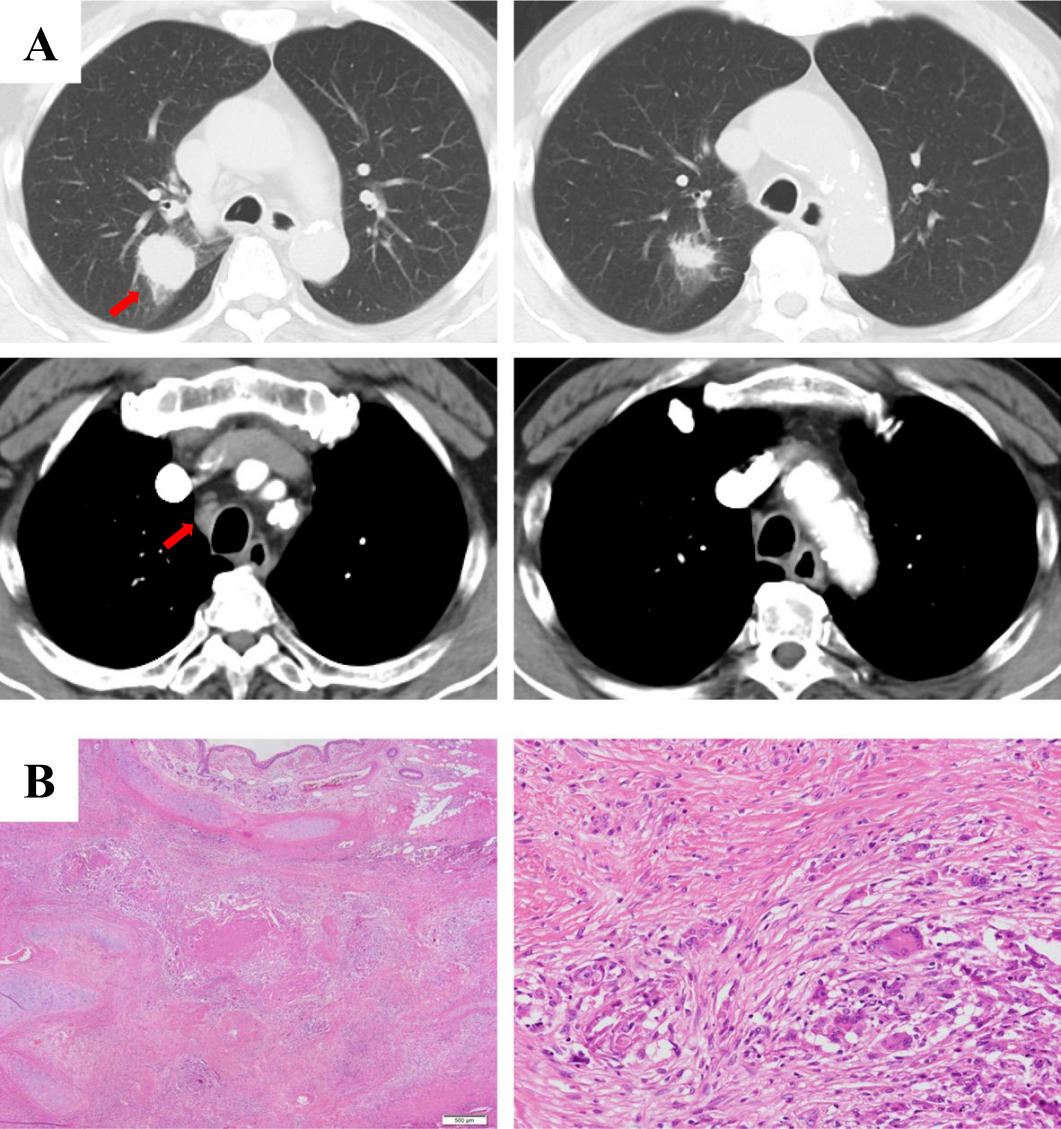
A representative case is shown (Case 4). **A** The left row is the results of a radiological evaluation by CT before treatment, and the right is the findings after induction CRT. The left upper row shows a primary lesion in the right upper lobe. The left lower row shows mediastinal lymph node (#4) swelling before treatment (clinical stage IIIA). The red arrows indicate the tumor and mediastinal lymph nodes. The upper right row shows mild regression of the primary lesion after induction CRT. The mediastinal lymph node (#4) shows also good regression in the right lower row (re-evaluated clinical stage IA3 after pretreatment). **B** The left row shows histological findings of the sections obtained from the surgical specimen, demonstrating fibrosis, foreign body reaction, and chronic inflammation with necrotic nests (original magnification, × 20). The right row shows the presence of lymphocytes, histiocytes, multinucleated giant cells, and fibroblasts in the fibrotic areas, but there are no remnants of viable cancer, suggesting a significant therapeutic effect (original magnification × 200)

Six hundred and sixty-one consecutive lung cancer patients underwent pulmonary resection in our hospital between 2020 and 2023. Twenty surgical patients had a history of prior treatment. Two patients underwent salvage surgeries. Therefore, 18 patients underwent induction chemotherapy followed by surgery. Among them, 15 patients underwent induction therapy including nab-PTX followed by surgery. These cases are summarized in Table 1.The mean BI and preoperative CEA values were 1057 and 9.1, respectively. These patients included 13 men and 2 women, with a median age of 67 (range, 54–77) years old. Seven patients were diagnosed with adenocarcinoma (AD) and six with squamous cell carcinoma (SQ). Pleomorphic carcinoma and anaplastic carcinoma were diagnosed in one case each. All patients had previously received CRT. In principle, concurrent CRT consisted of weekly administration of nab-PTX (50 mg/m^2^) plus CBDCA (AUC 2) and thoracic radiotherapy (50 Gy/25 fractions) [21]. Strictly speaking, 1 patient received 35 mg of nab-PTX, and 1 patient (Case 2) received thoracic radiotherapy (45 Gy). There were also 3 patients who received thoracic radiotherapy (60 Gy). The median total number of cycles of administration of nab-PTX (50 mg/m^2^) plus CBDCA was 5 (3–6). There were four patients who were forced to discontinue or reduce the dose due to adverse drug reactions. The clinical stages were as follows: IIB (*n =*1), IIIA (*n* = 12), and IIIC (*n =*2). The mean interval between the initial treatment and surgery was 57 days. The re-evaluated clinical stage after pretreatment was diagnosed as stage IA1 in one, IA3 in one, IB in two, IIB in seven, and stage IIIA in four patients. The disease control rate (DCR) was 100%, with 53% (8/15) showing partial response (PR) and 47% (7/15) showing stable disease (SD). Downstaging was observed in 73% (11/15) of patients on comparing the initial clinical stage and the re-evaluated clinical stage after concurrent CRT, whereas upstaging was documented in no patients (Fig. 1A). A representative case with downstaging is shown in Case 4 (Fig. 2A). None of the patients received adjuvant chemotherapy.

**Table 1:** Patient characteristics and summary of the perioperative factors for patients with induction followed by operation using nab-PTX

Case	Sex	Age	BI^a^	CEA^b^	Histol^c^	cStage^d^	ycStage^e^	Interval^f^	Adverse events
1	M	68	1920	1.8	AD	IIB	IIB	46	none
2	M	64	860	73.0	AD	IIIA	IIB	29	Liver disfunction
3	M	62	1200	3.4	SQ	IIIA	IIB	41	none
4	M	69	1920	4.3	SQ	IIIA	IA3	43	none
5	M	72	440	2.2	AD	IIIA	IA1	57	Leukopenia
6	M	72	0	5.7	AD	IIIA	IB	47	Leukopenia
7	F	77	1360	1.9	AD	IIIA	IIIA	44	Liver disfunction
8	F	54	600	1.6	SQ	IIIA	IIB	61	none
9	M	60	1000	1.9	Pleo	IIIC	IIIA	45	neutropenia
10	M	69	800	3.7	Anap	IIIA	IIIA	70	none
11	M	62	2040	3.8	SQ	IIIC	IIB	147	radiation pneumonitis, febrile neutropenia
12	M	67	1000	6.3	AD	IIIA	IIIA	84	none
13	M	75	1240	2.7	AD	IIIA	IB	69	none
14	M	60	680	4.7	SQ	IIIA	IIB	84	Liver disfunction
15	M	65	800	18.9	SQ	IIIA	IIB	98	none

BI^a^: Brinkman Index (calculated by multiplying the number of cigarettes smoked per day by the number of years that the subject had been smoking), CEA^b^, preoperative data of carcinoembryonic antigen, Histology^c^, AD, adenocarcinoma; SQ, squamous cell carcinoma; Pleo, pleomorphic carcinoma; Anap, anaplastic carcinoma, cStage^d^: clinical stage, ycStage^e^: re-evaluated clinical stage after pretreatment, Interval^f^: Interval between the initial treatment and surgery (days),

### Adverse events

Adverse drug reactions were observed in seven patients. Mild toxicities included liver dysfunction (G1: two patients, G2: one patient), leukopenia (G1: one patient, G2: one patient), and radiation pneumonitis (G2: one patient, Case 11). Febrile neutropenia (G3) was observed in one patient. The interval between the initial treatment and surgery for patients with radiation pneumonitis was 147 days (Case 11).

### Surgery

The surgical procedures were lobectomy (*n =*14) and bilobectomy (*n =*1), and pulmonary sleeve resection to avoid pneumonectomy was performed in 3 patients. Among these patients, concurrent pulmonary artery (PA) plasty was also performed in one patient (Table 2). The median operative time was 174 min, and the median blood loss was 40 ml. Complete resection was performed in all patients. The median number of days since discharge from the hospital after surgery was 10.

**Table 2:** Summary of the intraoperative factors of patients who underwent induction followed by operation using nab-PTX and relatively immediate data after surgery

Case	Surgical procedure^a^	Operative time (m)	Blood loss (ml)	Discharge^b^	Postoperative complication^c^
1	Sleeve RUL + LND + PA plasty	257	160	10	none
2	Sleeve RUL + LND	202	240	16	none
3	Sleeve RUL + LND	233	365	9	none
4	RUL + LND	174	70	9	none
5	RUL + LND	156	50	10	none
6	RUL + LND	107	5	13	air leak (3a)
7	RUL + LND	120	5	11	none
8	LLL + LND	135	10	5	none
9	LLL + LND	117	5	14	pneumonia (2)
10	LUL + LND	136	30	9	air leak (3a)
11	RUL + LND	164	50	12	none
12	LUL + LND	139	80	9	none
13	LUL + LND	132	20	7	none
14	LLL + LND	163	40	12	none
15	RMLL + LND	134	40	24	acute exacerbation of interstitial pneumonia (5)

Surgical procedure^a^, Discharge^b^: Number of days from surgery to discharge from hospital, Postoperative complication^c^: modified version of the Clavien-Dindo classification in parentheses *RUL* right upper lobectomy, *LLL* left lower lobectomy, *LUL* left upper lobectomy, *RMLL* right middle and lower lobectomy, *LND* lymph nodal dissection. *PA* pulmonary artery

### Postoperative complications

Postoperative complications occurred in four cases. Details of the postoperative complications are shown in Table 2. Two cases of air leak and one case of bacterial pneumonia were successfully managed. However, 1 patient developed acute exacerbation of interstitial pneumonia (IP) and died 24 days postoperatively despite intensive care (case 15). Thus, the 30-day mortality rate was 6.6%.

### Pathological outcomes

**Fig. 3:**
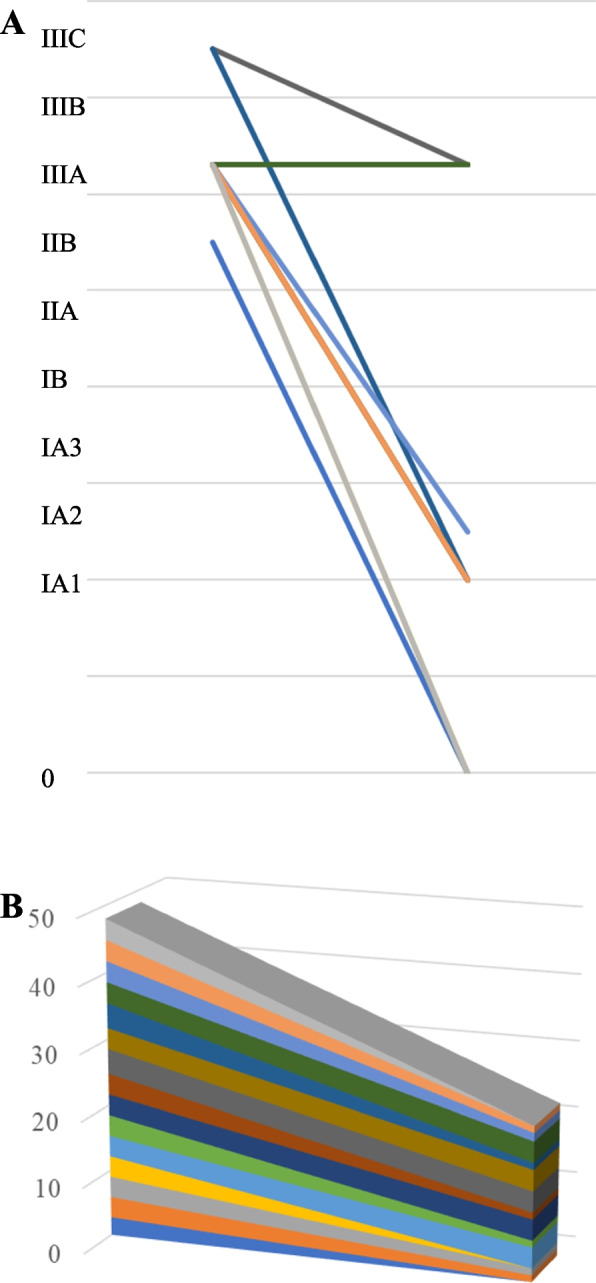
**A** The line chart of changes in the clinical stage and re-evaluated pathological stage after surgery in each patient. **B** A three-dimensional stacked area graph of changes in the pathological stage

Of the 16 patients who were diagnosed with positive lymph node metastases before treatment, only 5 cases were pathologically confirmed to be positive for lymph node metastasis after surgery. The re-evaluated pathological stage after pretreatment was diagnosed as stage 0 in 3 patients, stage IA1 in 6, IA 2 in 1, and stage IIIA in 5 (Table 3). The pathologic complete response (pCR) rate was 20.0%. Downstaging was observed in 73% (11/15) of patients (Fig. 3A) on comparing the initial clinical stage and re-evaluated pathological stage after surgery, whereas upstaging was documented in no patients.

**Table 3:** Summary of the perioperative factors of patients who underwent an induction followed by operation using nab-PTX

Case	ypstage^a^	Therapeutic effect^b^	Site of recurrence	RFS	OS
1	0	3	cervical lymph node	776	1225
2	IA1	2	brain meta	197	199
3	IA1	2	ipsilateral mediastinal lymph nodes	538	1382
4	0	3	none	1173	1173
5	IIIA	1a	none	186	186
6	IA1	2	none	808	808
7	IIIA	2	none	941	941
8	IA1	2	none	680	680
9	IIIA	1a	ipsilateral another lung lobe	1039	1613
10	IIIA	2	none	854	854
11	IA1	2	none	746	746
12	IIIA	1a	bilateral lung	151	260
13	IA2	2	none	275	275
14	IA1	2	none	270	270
15	0	3	none	24	24

ypstage^a^: Re-evaluated pathological stage after surgery, Therapeutic effect^b^, *Ef3* pathological complete response, *Ef2* pathological major response, *Ef1a* pathological extremely minor response

The pathological effects of previous therapy were as follows: Ef3 (*n =*3), Ef2 (*n =*9), and Ef1a (*n =*3). A representative case with a significant therapeutic effect is shown (Case 4: Fig. 2B). In total, 5 (33.3%) of the 15 patients developed disease recurrence after surgery. Regarding the sites of tumor recurrence, hematogenous metastases accounted for most instances of disease recurrence. The site of recurrence was the cervical lymph node, brain ipsilateral mediastinal lymph nodes, ipsilateral lung lobe, and bilateral lung metastasis in one case each. The median RFS and OS were 680 and 746 days, respectively.

### Prognoses and recurrent disease

The median follow-up duration was 746 days. At the last follow-up examination, nine patients were alive and free of cancer, four were alive with recurrent cancer, one had died of cancer, and one had died due to postoperative complications, as mentioned above.

## Discussion

Adverse drug reactions were observed in 7 patients (46.7%) in this series. All adverse events were relatively minor. Therefore, toxicity is tolerable and generally within the expected range. However, one patient died due to an acute exacerbation of IP after surgery. This patient had a short surgical time and small amount of blood loss but a history of IP. We concluded that there was no direct causal relationship with the toxicity of nab-PTX because no side effects were observed before the surgery. However, careful patient selection is required, depending on the surgical intervention itself.

Regarding downstaging, 11 cases (73%) in both proportions regarding the clinical and re-evaluated clinical stage, and clinical and re-evaluated pathological stage. In general, the maximum tumor diameter may not reflect the tumor size after neoadjuvant therapy [22]. Cascone et al. noted cases in which patients achieved stable disease radiographically but had major pathological response (MPR) or showed marked pathologic tumor regression at surgery. Furthermore, in some cases, they noted the radiographic appearance of nodal disease progression with enlargement and/or an increased fluorodeoxyglucose (FDG) uptake in the nodes on CT and PET-CT restaging scans, respectively, after neoadjuvant ICI administration. However, an invasive pathologic examination of flaring nodes revealed changes in immune cell infiltration but not malignancy [22]. Liu et al. reported that when sufficient cycles of neoadjuvant therapy are completed, many types of tumor necrosis, tissue fibrosis, and inflammatory response contribute to maintaining the tumor bulk [23], which also causes discrepancies in images and pathology [24]. In fact, the slope of the three-dimensional stacked area graph between clinical and re-evaluated clinical stages and clinical and re-evaluated pathological stages was different; the slope of the former emphasizes the therapeutic effect of induction therapy, including nab-PTX, compared to that of the latter.

A pathologic response after neoadjuvant chemotherapy plus or minus radiotherapy is associated with significant improvements in the survival of patients with resectable NSCLC based on a meta-analysis [25]. Thus, the pathologic complete response (pCR) and MPR rates have been proposed as surrogate endpoints [26]. We previously reported that the proportion of patients with Ef2/3 was 56% in induction CRT with PTX for resectable LA-NSCLC [10]. The proportion of patients with Ef2/3 was 80% (12/15) in the present cohort. Of note, the therapeutic effect of induction therapy, including nab-PTX, in our series was relatively promising. The discrepancy between 56 and 80% may be due to the structure of the drug and, ultimately, the difference in tissue permeability of the tumor by nab-PTX. In fact, downstaging was observed in 73% of patients in the present study. The Southwest Oncology Group trial showed that only 15 (9%) of 169 patients randomly assigned to receive preoperative PTX and CBDCA followed by surgical resection were found to have pCR [7]. On reviewing the three-dimensional stacked area graph of changes, the slope of change was larger in the clinical stage and re-evaluated pathological stage than in the clinical stage and re-evaluated clinical stage (Figs. 1B, 3B). This suggests that a pathological evaluation after surgery is more effective than imaging.

1. It presumably is more effective to use nab-PTX than PTX as a partner cytotoxic drug with platinum-based chemotherapy for LA-NSCLC. Chemotherapy may elicit anticancer immunity through the release of potentially immunogenic tumor antigens [27]. The combination of an ICI and nab-PTX is therefore an interesting regimen in the induction setting before surgery and may also hold promise for resectable LA-NSCLC. In fact, Liu et al. reported that 42 patients (53.2%) with pCR or MPR among 79 with neoadjuvant chemo-immunotherapy had received regimens containing nab-PTX [24]. Two recent phase II trials using an ICI (a monoclonal antibody against PD-1 plus nab-PTX based chemotherapy) showed good MPR rates in the 60% range [28, 29]. Preoperative treatment with immune checkpoint inhibitors (ICIs) has also shown good results [22, 24] but was not included in this case. From the perspective of local recurrence, chemoradiotherapy has an advantage due to its high pCR [9, 10, 22, 24]. However, the appearance of radiation lung damage in the late stage is also possible. Therefore, there are currently no obvious selection criteria. However, if the radiation treatment range is quite wide, such as metastasis of the upper mediastinal lymph node of lung cancer in the lower lobe, ICIs may be selected for preoperative treatment. Conversely, patients with immune abnormalities such as endocrine diseases are likely to develop AEs to ICIs, so CRT will be chosen for preoperative treatment.

The present study had several limitations that warrant mention, including its retrospective design, small number of patients, and the fact that it was conducted at a single institution. There was also patient selection bias, with a short median follow-up time. Despite these limitations, induction therapy, including nab-PTX followed by surgical resection, has been associated with an encouraging therapeutic effect. Accordingly, induction CRT, including nab-PTX, followed by resection, may be a viable alternative treatment that offers the chance to achieve a cure in carefully selected cases.

## Conclusion

The therapeutic effect of induction therapy including nab-PTX was promising. Induction CRT, including nab-PTX, followed by resection, may be a viable alternative treatment option for locally advanced NSCLC.

## Data Availability

No datasets were generated or analysed during the current study.

## Abbreviations

NSCLC: Non-small cell lung cancer
CBDCA: Carboplatin
nab-PTX: Nab-paclitaxel
LA-NSCLC: Locally advanced NSCLC
CRT: Chemoradiation therapy
AUC: Area under the plasma concentration time curve
ICI: Immune checkpoint inhibitor
PFS: Progression-free survival
PTX: Paclitaxel
CT: Computed tomography
ECG: Electrocardiography
UCG: Ultrasound cardiography
PET: Positron emission tomography
MRI: Magnetic resonance imaging
BI: Brinkman Index
VATS: Video-assisted thoracoscopic surgery
IASLC: International Association for the Study of Lung Cancer
Ef3: Pathological complete response
Ef2: Pathological major response
Ef1: Pathological minor response
Ef0: Pathological no response,
OS: Overall survival
RFS: Recurrence free survival
AD: Adenocarcinoma
SQ: Squamous carcinoma
DCR: Disease control rate
PR: Partial response
SD: Stable disease
PA: Pulmonary artery
IP: Interstitial pneumonia
MPR: Major pathological response
FDG: Fluorodeoxyglucose
pCR: Pathological complete response
